# Protective Effects of Cereal-Based Fermented Beverages Against 5-Fluorouracil-Induced Intestinal Damage in Mice

**DOI:** 10.3390/nu16244332

**Published:** 2024-12-16

**Authors:** Dongze Qin, Wenhui Fu, Yi Sun, Lingda Zhao, Haiwei Liu, Dancai Fan, Dongfei Tan, Xuemeng Ji, Shuo Wang

**Affiliations:** 1 Tianjin Key Laboratory of Food Science and Health, School of Medicine, Nankai University, Tianjin 300071, China; qindongze0601@163.com (D.Q.); ahfyfwh@163.com (W.F.); 9920220161@nankai.edu.cn (Y.S.); 2120201373@mail.nankai.edu.cn (L.Z.); haiweiliu927@163.com (H.L.); fandancai@nankai.edu.cn (D.F.); jixuemeng@nankai.edu.cn (X.J.); 2 Institute of Agro-Product Safety and Nutrition, Tianjin Academy of Agricultural Sciences (TAAS), Tianjin 300192, China; tandongfei1991@163.com

**Keywords:** cereal, fermentation, 5-flourouracil, intestinal mucositis, oxidative stress

## Abstract

Background: 5-Fluorouracil (5-FU) is a common chemotherapeutic medication used to treat cancer. However, the intestinal tract may sustain oxidative damage as a result. Objectives: The purpose of this study was to clarify the underlying molecular mechanisms and examine the preventive benefits of cereal-based fermented drinks (CFBs) against intestinal injury in mice caused by 5-FU. Methods: The mice were injected intraperitoneally with 5-FU to induce intestinal mucosal and treated with CFB. The factors for intestinal barrier integrity, oxidative stress and inflammation were measured. Results: The findings demonstrated that CFBs had high levels of polyphenol, flavonoids, and peptides and had in vitro high free radical scavenging capacity. Furthermore, CFBs effectively ameliorated 5-FU-induced intestinal epithelium damage, characterized by increasing intestinal tight junctions and reducing apoptosis in intestinal cells. These protective effects may attribute to the increased activity of antioxidant-related enzymes (SOD, CAT, and GSH) as well as decreased amounts of inflammatory and oxidative damage markers (IL-1β, TNF-α, and MDA) in the intestinal tract. Conclusions: Overall, these results show that CFBs can mitigate intestinal damage caused by 5-FU by reducing oxidative stress, suggesting the potential utility of CFBs for therapeutic treatment against intestinal mucositis.

## 1. Introduction

According to data from the International Agency for Research on Cancer, approximately 10.3 million people died from cancer and there were 19.3 million new diagnoses globally in 2020. Cancer is the disease with the highest mortality rate and its incidence continues to rise. Currently, chemotherapy is the primary treatment used for cancer. 5-FU is a typical chemotherapeutic medication used for the treatment of gastric, colon, breast, and other cancers. Similar to uracil, it is a heterocyclic aromatic organic molecule that has a fluorine atom at position C-5 rather than a hydrogen atom. Because it resembles a pyrimidine, it functions as an antimetabolite and is readily integrated into DNA and RNA to replace uracil or thymine [1,2]. Therefore, by blocking intracellular thymidylate synthetase from converting deoxynucleoside to thymidinase, it can disrupt DNA synthesis and cause apoptosis [3,4]. However, 5-FU is associated with numerous side effects during anticancer therapy, including fatigue, loss of appetite, and diarrhea. This may have a substantial impact on the patient’s life as well as the efficacy of cancer therapy [5,6,7].

The excessive generation of reactive oxygen species (ROS) in tissues is known as oxidative stress. It can cause cellular destruction [8]. 5-FU exerts its therapeutic effects by inducing oxidative stress in cancer cells. However, during 5-FU treatment, the resulting ROS in the intestines can damage intestinal epithelial cells, increase pro-inflammatory factors, and reduce antioxidant secretion, ultimately leading to intestinal oxidative damage and inflammation, which can cause gastrointestinal mucositis [9,10,11,12]. Currently, the intestinal damage caused by chemotherapy can only be alleviated, with limited success in reducing its duration and severity [13]. Therefore, dietary intervention is of great significance in preventing and treating intestinal oxidative damage during chemotherapy.

It has been demonstrated that broomcorn millet, millet, sorghum rice, waxy rice, coix seed, and other herbal medicines and food homology cereals can improve human health, illness resistance, and physiological balance [14,15,16,17]. Furthermore, research has shown that fermentation can improve the functionality of these cereals by degrading polysaccharides and proteins, as well as releasing bound polyphenols and bioactive peptides. For example, when Antognoniet al. fermented wheat using several strains of lactic acid bacteria, the phenolic compounds were released, which enhanced the antioxidant activity of wheat [18]. Similarly, when Mohapatra et al. fermented sorghum grains, they observed an increase in the level of amino acids, as well as a decrease in anti-nutritional substances, such as trypsin inhibitors. This indicated that fermentation with lactic acid bacteria could improve nutrient bioavailability in fermented sorghum [19]. Bioactive peptides obtained from fermented cereals like wheat, rye, and barley and the pseudo-cereal amaranth have also demonstrated antioxidant and anti-inflammatory properties [20,21,22,23]. Moreover, research has shown that yam, ginseng, *Lycium barbarum*, and cinnamon, which are included in the list of herbal medicines and food homology, can enhance immunity and protect the gastrointestinal tract. Suitable fermentation conditions can facilitate the release of these herbal medicines’ active components and transform them into new bioactive compounds [24,25]. However, the limited fermentable nitrogen and carbon sources in herbal medicines, along with their antimicrobial components, can restrict the fermentative activities of the strains. Consequently, combining cereals with herbal medicines can improve the bacterial strains’ capacity to ferment and increase the functionality of the fermentation products.

In this study, five types of cereal raw materials (black glutinous rice, fragrant rice, rice, broomcorn millet, and millet) were selected for their ability to release peptides with gastrointestinal regulatory capacity during fermentation. These cereals were then mixed with berries and herbal medicines with functional properties, including immunomodulation, bone marrow hematopoiesis, and gastrointestinal protection. A combination of commercial strains of lactic acid bacteria (*Lactobacillus bulgaricus* and *Streptococcus thermophilusand* inside) and yeast (baker’s yeast and liqueur yeast) was introduced to ferment cereal-based beverages. Subsequently, we investigated the potential of cereal-based fermented beverages (CFBs) to prevent gastrointestinal mucositis caused by 5-FU in vivo, along with the possible molecular pathways contributing to these protective effects.

## 2. Materials and Methods

### 2.1. Preparation of CFBs

Five types of selected grain raw materials (black glutinous rice, fragrant rice, rice, broomcorn millet, and millet at a 1:1:1:1:1 ratio) were mixed with sterilized distilled water at a ratio of 2:1 (*w*/*v*) and soaked for 60 min. Following this, they underwent steam sterilization and ripening at 100 °C for 60 min. After the grain was cooled to room temperature, they were then mixed with either 3% (*m*/*m*) herbal medicines powder (yam, ginseng, *Lycium barbarum*, cinnamon, and blueberry at a 1:1:4:6:2 ratio) or without. Following this, 1% (*m*/*m*) commercial lactic acid bacteria (*Lactobacillus bulgaricus* and *Streptococcus thermophilusand* inside), baker’s yeast, and liqueur yeast (1:2:1 ratio, sourced from China Angel Yeast Co., Ltd. (Yichang, China)) were added. After fermentation for 7 days at 30 °C, the samples were filtered and subjected to ultra-high temperature sterilization treatment at 135 °C for 8 s. The resulting supernatant of the samples with herbal medicines was denoted as ZLCM, and the resulting supernatant of the samples without herbal medicines was ZLC. A commercial cereal-based juice served as a positive control (JGJZ).

### 2.2. Quality Analysis of CFBs

#### 2.2.1. Assessment of Total Phenolic Content (TPC)

To assess TPC, the Folin–Ciocalteu technique was utilized [26]. In summary, 200 µL of the sample (diluted 5 times with distilled water) was extracted, and then, 2.5 mL of the Folin–Ciocalteu reagent was applied. Following this, the addition of distilled water produced the final amount of 10 mL, and then, 2 mL of sodium carbonate solution was added. Following a thorough mixing, the mixture was left at 25 ± 2 °C for 15 min. Next, the resultant solution’s absorbance at 760 nm was measured. TPC readings were expressed in milligrams per milliliter (mg/mL) as gallic acid equivalents after being computed against gallic acid.

#### 2.2.2. Assessment of Total Flavonoid Content (TFC)

To determine TFC, the aluminum chloride colorimetric technique was conducted [27].

In short, a 5% NaNO_2_ liquid (0.3 mL) was mixed with 100 µL of the extract, which had been carefully diluted 5 times with distilled water. A 10% Al(NO_3_)_3_ (0.15 mL) liquid was applied after 6 min of incubation; then, 5 min were allowed for the mixture to react. The liquid was then mixed with 2.0 mL of 1 M NaOH. After 10 s of vigorous vortexing, dehydrated alcohol was used to dilute the contents to a final amount of 10 mL. Shortly later, utilizing a spectrophotometer, the absorbance at 510 nm was measured. The findings were measured in milligrams of rutin equivalent per gram of dry weight (mg/mL).

#### 2.2.3. Protein Content and Molecular Weight (MW) Distribution

Using a standard calibration curve with bovine serum albumin, the protein content was determined. Utilizing a microplate reader, the absorbance at 595 nm was measured after the sample (20 μL) and Bradford reagent (200 μL) were mixed for 5 min.

Following the previously established method [28], the MW distribution of CFBs was analyzed using HPLC (Waters e2695, Milford, MA, USA) and Waters 2489 UV detector (Milford, MA, USA). A TSK Gel G2000 SWXL column (7.8 mm × 300 mm; particle size 7 µm, TOSOH Co., Ltd., Tokyo, Japan) was used. The rate of flow was set at 0.5 mL/min, and the mobile phase was composed of acetonitrile, water, and trifluoroacetic acid in a proportion of 45:55:0.1 (*v*/*v*/*v*). Measurements of absorbance were made at 214 nm in wavelength.

### 2.3. Chemical Antioxidant Capacity of CFBs

#### 2.3.1. The Radical Scavenging Activity of 2,2-Diphenyl-1-picrylhydrazyl (DPPH)

Using a previously published technique, the CFBs’ ability to scavenge DPPH radicals was examined [29]. The sample and a 0.2 mM DPPH solution in ethanol were combined for 30 min at 25 ± 2 °C in a dark environment. At 517 nm, the combination’s absorbance was measured. Using the following formula, the DPPH scavenging effect was determined:

$$\mathrm{scavenging}\;\mathrm{rate}\;(\%)=\left(1-\frac{A_{2}-A_{1}}{A_{0}}\right)\times 100$$

In this case, the absorbance of the mixture containing the test sample solution is A_2_, the absorbance of the mixture containing the sample replaced by dehydrated alcohol is A_1_, and the absorbance of the mixture having the sample replaced by ultrapure water is A_0_.

#### 2.3.2. The Radical Scavenging Activity of 2,2′-Azino-bis (3-ethylbenzothiazoline-6-sulfonic acid) Diammonium Salt (ABTS)

For the ABTS test, 7.4 mM ABTS and 2.6 mM potassium persulfate were combined in an equal amount and left for 12 h at 25 ± 2 °C in a dark environment. Then, 100% methanol was used to dilute the mixture 50 times. The absorbance was measured at 734 nm after the sample (0.6 mL) and diluted ABTS solution (1.6 mL) reacted for 7 min at 25 ± 2 °C. The formula below was used to calculate ABTS’s scavenging effect:

$$\mathrm{scavenging}\;\mathrm{rate}\;(\%)=\left(\frac{A_{0}-A}{A_{0}}\right)\times 100$$

In this case, the mixture’s absorbance with the test sample solution is A, and the mixture’s absorbance with the sample substituted by dehydrated alcohol is A_0_.

### 2.4. Mouse Model of Intestinal Injury

**Figure 1:**
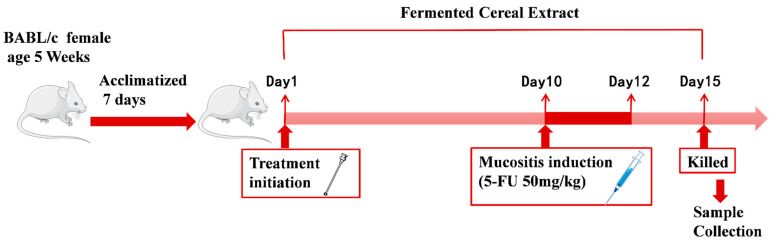
The scheme of animal experiments: BALB/c mice (*n* = 10 in each group) were treated with CFB for 15 days. On the 10th day, intestinal injury was induced by intraperitoneal (i.p.) injection of 5-FU (300 mg/kg).

Following the Chinese government’s Guidelines for Keeping Experimental Animals, healthy female SPF BALB/c mice, aged 5 weeks, were used in all studies. They were acquired from Beijing Vital River Laboratory Animal Technology Co., Ltd. (Beijing, China) and kept in the cages in the animal room at Nankai University Laboratory Animal Center (permission number: SYKX 2019-0001) under controlled conditions (temperature, 20–25 °C; humidity, 50–60%; 12/12 h light/dark cycle). The mice were randomly divided into five groups after acclimation (10 mice each, for a total of 50 animals) and administered the following treatments: Ctrl (control group), 5-FU (model group), JGJZ (intragastric administration with 300 μL JGJZ per day), ZLC (intragastric administration with 300 μL ZLC per day), and ZLCM (intragastric administration with 300 μL ZLCM per day). A single intraperitoneal (i.p.) dose of 5-FU (300 mg/kg) caused intestinal mucositis from day 10 to 12, except for the Ctrl group [30]. Throughout the study, the mice had unrestricted access to food and water. After 15 days of treatment, based on animal welfare principles, all mice were anesthetized and cervical dislocated for collecting serum, spleen, small intestine, and ileum for further analysis (Figure 1).

### 2.5. Clinical Index Examination

When the experiment was over, the hair surrounding the anus and buttocks were observed. After collecting blood samples, in order to retrieve tissues from the small intestine, ileum, and spleen for further morphological and biological analyses, the animals were subsequently put to death by cervical dislocation.

### 2.6. Analysis of Intestinal Histology and Morphology

After euthanizing the mice, all of the small intestine was removed and examined, after which 2 to 3 cm of ileum was taken for histological examination. Following a 24 h immersion in 10% buffered formaldehyde, the intestine tissue was placed in paraffin wax. Hematoxylin and eosin (HE) and periodic acid Schiff (PAS) were used to stain sections (4 μm) that had been placed on glass slides. Under an optical microscope, tissue damage in the mouse ileum was evaluated using HE staining. Villus height measurements (from villus tip to villus–crypt junction) per intestinal tissue section were measured, and values are presented as averages. Goblet cells were examined in samples stained with PAS. Ileum tissue apoptotic cells were measured by TUNEL staining.

### 2.7. Quantification of Gene Expression

Following the manufacturer’s directions, the total RNA was extracted from small intestinal tissue using TRIzol Reagent (Ambion, Austin, TX, USA). The extracted RNA was then reverse-transcribed into cDNA using the Revert Aid First Strand cDNA Synthesis Kit (Thermo Fisher Scientific, Waltham, MA, USA). The mRNA expression levels of intestinal damage, oxidative damage, and apoptosis genes were identified using qRT-PCR on the CFX Connect Real-Time System (BIO-RAD, Hercules, CA, USA). Table 1 lists the primers that were utilized. By comparing the target gene to β-actin and computing the results using the 2^−∆∆Ct^ method, the gene’s relative quantity was determined.

### 2.8. Intestine Tissue Biochemical Analysis

The contents of MDA, DAO, GSH, SOD, and CAT in small intestine tissue were determined using commercial test kits (Nanjing Jiancheng Bioengineering Institute, Nanjing, China). IL-1β and TNF-α levels were assessed using ELISA kits (Jiangsu Meibiao Biotechnology Co., Ltd. (Nanjing, China)).

### 2.9. Statistical Analysis

The GraphPad Prism 9.5.0 software (GraphPad, San Diego, CA, USA) was used to calculate the effects of CFBs on the observable variables. The results are presented as means ± SEM. Differences between the experimental and control groups were ascertained using *t*-tests. Differences were regarded as statistically significant at a *p*-value < 0.05 (* *p* < 0.05, ** *p* < 0.01, *** *p* < 0.001, or **** *p* < 0.0001).

## 3. Results

### 3.1. The Quality and Chemical Antioxidant Activity of CFBs

**Figure 2:**
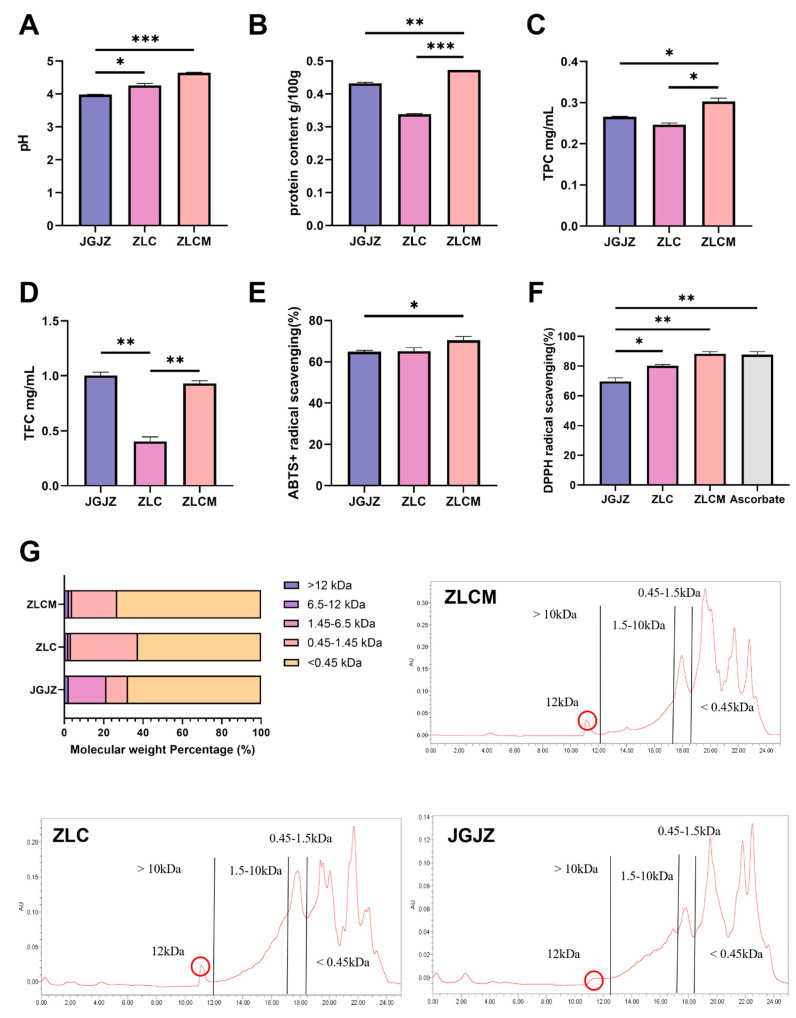
The quality and chemical antioxidant activity of CFBs. ZLC: the fermentation supernatant of samples without herbal medicines; ZLCM: the fermentation supernatant of samples with herbal medicines; JGJZ: the commercial cereal-based juice; Ascorbate: the positive control group (Vitamin C). (**A**) pH values; (**B**) protein content; (**C**) TPC; (**D**) TFC; (**E**) ABTS radical scavenging activity; (**F**) DPPH radical scavenging activity; (**G**) molecular weight distribution (The red-circled part is the elution peak of the 12 kDa molecular weight protein). Data are presented as mean ± SEM, and differences were regarded as statistically significant at a *p*-value < 0.05 (* *p* < 0.05, ** *p* < 0.01, or *** *p* < 0.001).

The ZLC and ZLCM samples had much higher pH values than the JGJZ sample (*p* < 0.05 and *p* < 0.001, respectively) (Figure 2A,B). The protein content of the ZLCM sample was considerably higher than that of the JGJZ and ZLC samples (*p* < 0.01 and *p* < 0.001, respectively). As revealed in the HPLC chromatograms (Figure 2G), the low MW content (MW < 1.45 kDa) in the ZLC and ZLCM samples was higher than that in the JGJZ sample. Moreover, the MW content above 12 kDa and below 0.45 kDa in the ZLCM sample was higher than in the ZLC sample.

Furthermore, as demonstrated in Figure 2C, the TPC of ZLCM was higher than those of ZLC and JGJZ (*p* < 0.05 and *p* < 0.05, respectively). The TFC was the lowest in ZLC compared to the ZLCM and JGJZ samples (*p* < 0.01), while ZLCM and JGJZ showed similar TPCs with no significant difference (Figure 2D). According to the findings of the determination of the antioxidant peptide types (Table S1) and radical scavenging abilities (Figure 2E,F) of the CFBs, there were 77 types of antioxidant peptides in ZLC, 32 types in ZLCM, and 43 types in JGJZ. Additionally, compared to ZLC and JGJZ, ZLCM had a stronger capacity to scavenge ABTS radicals, though it was not statistically significant. The ZLCM and ZLC samples exhibited stronger DPPH radical scavenging activity (*p* < 0.01 and *p* < 0.05, respectively) than the JGJZ sample.

### 3.2. Clinical Index

**Figure 3:**
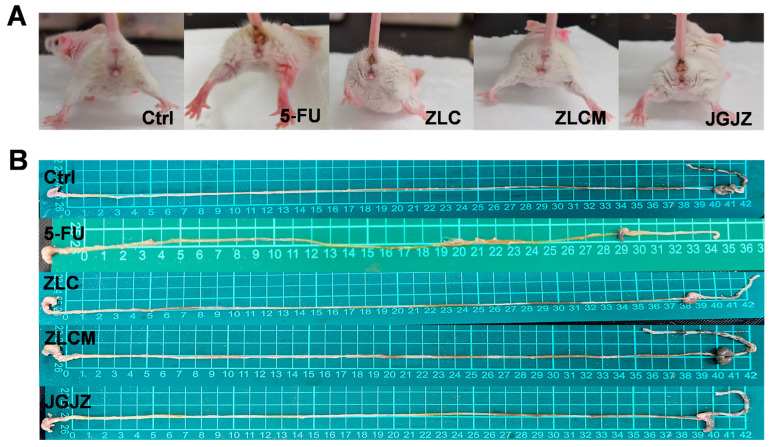
Clinical index of mice. (**A**) The contamination of the hair around the anus and buttocks; (**B**) The length of the small intestine. Ctrl: the control group; 5-FU: the model group; ZLC: pretreatment with ZLC; ZLCM: pretreatment with ZLCM; JGJZ: the positive control (pretreatment with JGJZ).

Mice in the 5-FU group had much more hair contamination around their anuses and buttocks than the Ctrl group’s mice (Figure 3A). The 5-FU group’s small intestine was shorter (Figure 3B). CFB was effective in preventing the reduction in intestinal length and mitigating the intestinal damage brought on by 5-FU therapy.

### 3.3. CFB Supplementation Ameliorated Immunosuppression in Mice Induced by 5-FU

**Figure 4:**
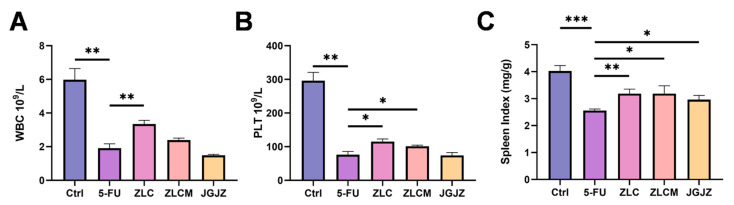
CFB supplementation ameliorated immunosuppression in mice induced by 5-FU. Ctrl: the control group; 5-FU: the model group; ZLC: pretreatment with ZLC; ZLCM: pretreatment with ZLCM; JGJZ: the positive control (pretreatment with JGJZ). (**A**) White blood cell (WBC); (**B**) platelet (PLT); (**C**) spleen index. Data are presented as mean ± SEM, and differences were regarded as statistically significant at a *p*-value < 0.05 (* *p* < 0.05, ** *p* < 0.01, or *** *p* < 0.001).

Immune organs are essential for activating the immune system and regulating immune activity [31]. To a certain degree, the spleen indexes could represent an animal’s immune function. As shown in Figure 4C, spleen index was significantly lower in the 5-FU group than in the Ctrl group (*p* < 0.0001). In the ZLCM, ZLC, and JGJZ groups, the spleen indexes were considerably elevated, particularly in the ZLC group (*p* < 0.01). However, no notable variations were seen between the samples. In addition, the human bone marrow is a hematopoietic organ and the fundamental immunological organ of humans, as well as the only source of blood cells [32]. In this study, WBC (*p* < 0.01) and PLT (*p* < 0.05, *p* < 0.05) counts dramatically dropped in the 5-FU group (Figure 4A,B). Nevertheless, CFBs could considerably raise the number of WBCs and PLTs after treatment with 5-FU. To sum up, these findings show that CFBs could reduce the bone marrow suppression and immunosuppression brought on by 5-FU (*p* < 0.01, *p* < 0.05), and it had better effects than JGJZ.

### 3.4. CFB Ameliorated 5-FU-Induced Intestinal Barrier Injury and Histopathological Changes

**Figure 5:**
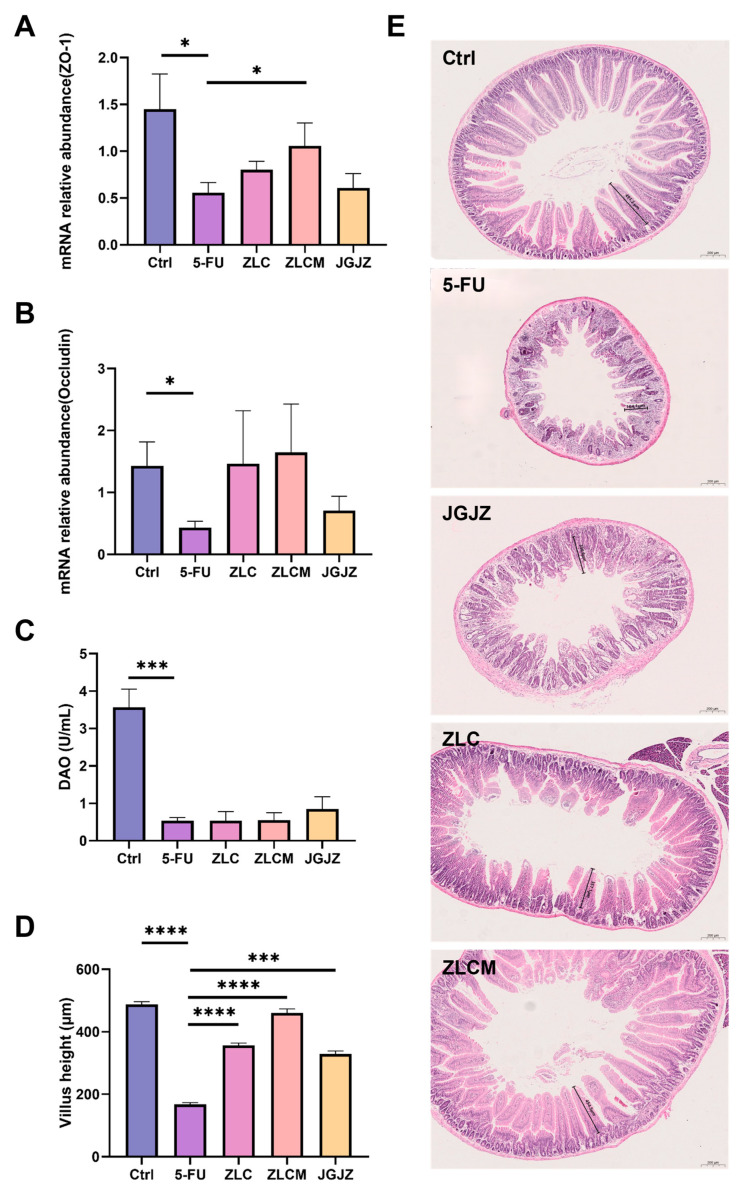
CFB ameliorated 5-FU-induced intestinal barrier injury and histopathological changes. Ctrl: the control group; 5-FU: the model group; ZLC: pretreatment with ZLC; ZLCM: pretreatment with ZLCM; JGJZ: the positive control (pretreatment with JGJZ). The mRNA expression levels of (**A**) ZO-1; (**B**) occludin; (**C**) DAO; (**D**) morphometric analysis of villus height; (**E**) histopathological sections of stained mucosal (5× objective, scale bar = 200 μm). Data are presented as mean ± SEM, and differences were regarded as statistically significant at a *p*-value < 0.05 (* *p* < 0.05, *** *p* < 0.001, or **** *p* < 0.0001).

To maintain immunological homeostasis and intestinal function, the gastrointestinal barrier is essential. It consists mostly of intestinal epithelial cells, tight junctions (TJs), and the gut microbiota [33]. The integrity and maturation of the small intestine are associated with plasma diamine oxidase (DAO) activity [34]. In our research, the degree of mucosal injury in mice following 5-FU administration was evaluated using changes in plasma DAO activity. In mice administered 5-FU, DAO activity dramatically decreased (*p* < 0.0001), suggesting that 5-FU could significantly damage the intestinal mucosa (Figure 5C). The ZLC and ZLCM groups had a greater DAO activity, but the difference did not reach statistical significance.

According to earlier research, one of the primary factors of barrier degradation is the loss of TJs. Accordingly, the mRNA expression of TJ proteins was evaluated in this study. According to the results, CFB treatment increased their expression levels, particularly the levels of ZO-1 in the ZLCM group (*p* < 0.05) (Figure 5A,B). These findings show that CFBs could regulate the expression of TJs, which were critical indicators of intestinal barrier integrity.

HE staining (Figure 5E) showed that the intestinal tissue structure in the Ctrl group was intact (496.7 ± 8.78 μm), while the 5-FU group had serious damage and the height of the villi (164.1 ± 5.03 μm) was obviously shorter (*p <* 0.0001). CFB treatment restored the morphology and height of the villi in the ileal tissue (ZLC: 357.1 ± 7.80 μm; ZLCM: 454.5 ± 12.78 μm; JGJZ: 336.6 ± 9.31 μm) (*p* < 0.0001, *p* < 0.0001, and *p* < 0.001, respectively), with a neat arrangement and relatively clear and intact structure of each layer (Figure 5D). Additionally, the ZLCM and ZLC groups were more effective than the JGJZ group.

### 3.5. Effects of CFBs on the Integrity of Intestinal Mucosal Barrier in Mice

**Figure 6:**
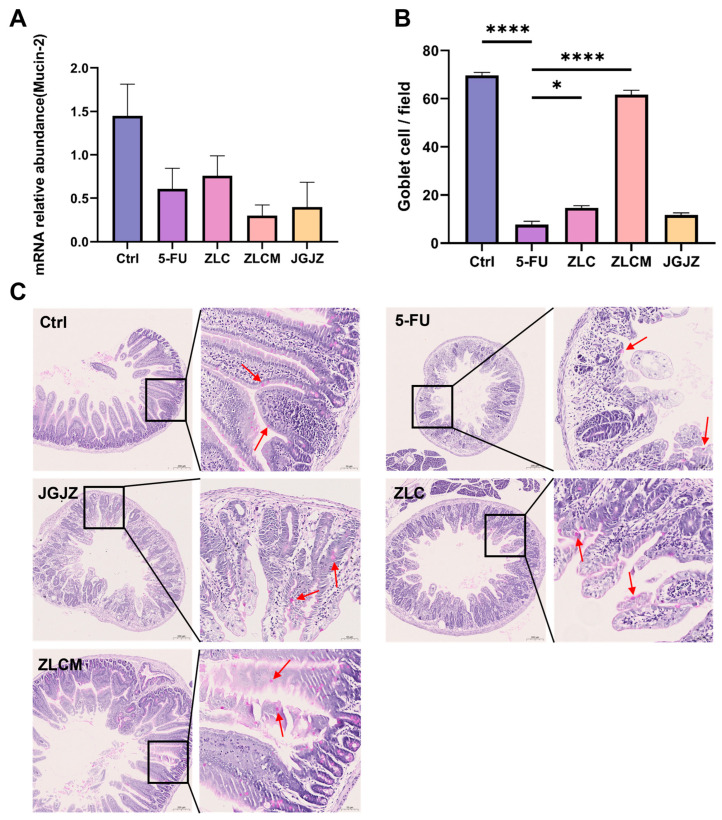
Effects of CFBs on the integrity of intestinal mucosal barrier in mice. Ctrl: the control group; 5-FU: the model group; ZLC: pretreatment with ZLC; ZLCM: pretreatment with ZLCM; JGJZ: the positive control (pretreatment with JGJZ). (**A**) The mRNA expression levels of Mucin-2; (**B**) number of goblet cells/field for experimental groups; (**C**) Alcian blue-periodic acid Schiff (AB-PAS) staining of ileum; the purple-magenta particles indicated by the arrow are goblet cells (20× objective, scale bar = 50 μm). Data are presented as mean ± SEM, and differences were regarded as statistically significant at a *p*-value < 0.05 (* *p* < 0.05, or **** *p* < 0.0001).

In order to prevent the bacterial and viral invasion of the epithelial surface, the intestinal mucosal barrier is necessary. Goblet cells, responsible for mucin secretion, are key components in epithelial protection, along with trefoil factor [35]. The main material in the goblet cells is mucopolysaccharide. In PAS staining, periodic acid oxidizes the hydroxyl groups on adjacent carbons of sugars into aldehyde groups, which then react with Schiff agent to turn purple-magenta. Therefore, after PAS staining, the goblet cell morphology in the small intestine tissue is purple-magenta. Our study mainly observed the distribution and quantity of purple-magenta particles, namely the neutral mucopolysaccharide material in goblet cells in the PAS staining results. As shown in Figure 6A–C, mice administered 5-FU showed a substantial reduction in small intestinal goblet cells (*p* < 0.0001). Mucin-2 expression decreased in the 5-FU group. However, mice in the ZLC and ZLCM groups had considerably more goblet cells in their intestines (*p* < 0.05 and *p* < 0.0001, respectively). Meanwhile, the Mucin-2 expression level was higher in the ZLC group. However, there was no discernible change. These results suggest that CFBs may reduce intestinal mucosal damage brought on by 5-FU while preserving intestinal mucosal barrier integrity.

### 3.6. CFB Reduced 5-FU-Induced Apoptosis of Intestinal Cells

**Figure 7:**
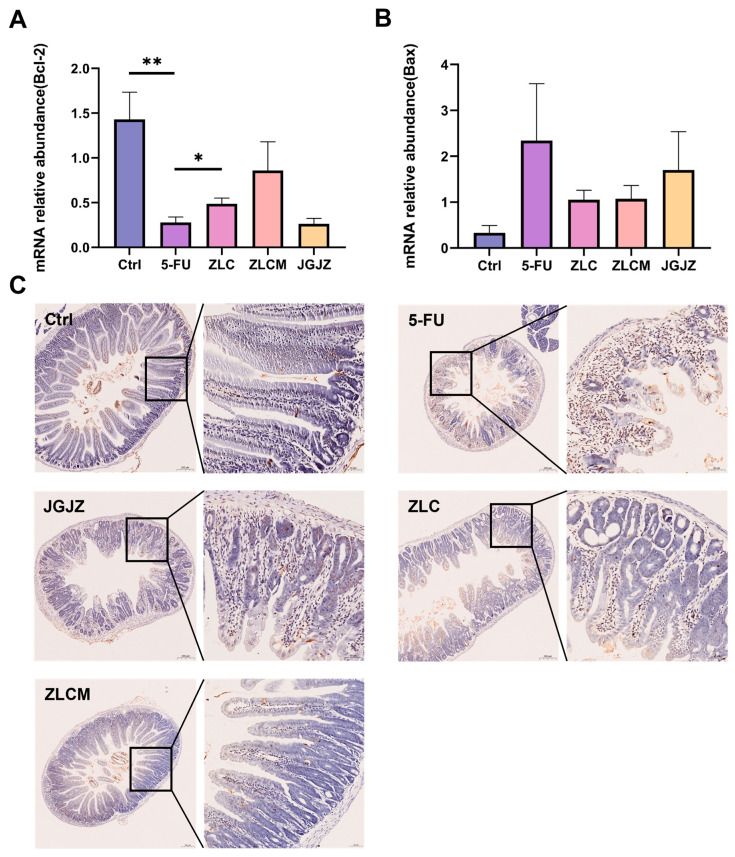
CFBs reduced 5-FU-induced apoptosis of intestinal cells. Ctrl: the control group; 5-FU: the model group; ZLC: pretreatment with ZLC; ZLCM: pretreatment with ZLCM; JGJZ: the positive control (pretreatment with JGJZ). (**A**) The mRNA expression levels of Bcl-2; (**B**) mRNA expression levels of Bax; (**C**) TUNEL staining (20× objective, scale bar = 50 μm). Data are presented as mean ± SEM, and differences were regarded as statistically significant at a *p*-value < 0.05 (* *p* < 0.05, or ** *p* < 0.01).

TUNEL staining, as shown in Figure 7C, revealed increased cell apoptosis in the 5-FU group. Conversely, CFB treatment remarkably reduced apoptosis. In order to directly show that 5-FU could lead to intestinal apoptosis in mice, our research evaluated the Bcl-2 and Bax gene expression levels. As shown in Figure 7A,B, 5-FU therapy increased Bax expression, while decreasing the mRNA expression level of the Bcl-2 gene (*p* < 0.01). CFB pretreatment inhibited the expression of genes linked to apoptosis, specifically raising the Bcl-2 level in the ZLC group (*p* < 0.05).

### 3.7. CFB Attenuated 5-FU-Induced Intestinal Oxidative Stress Injury

**Figure 8:**
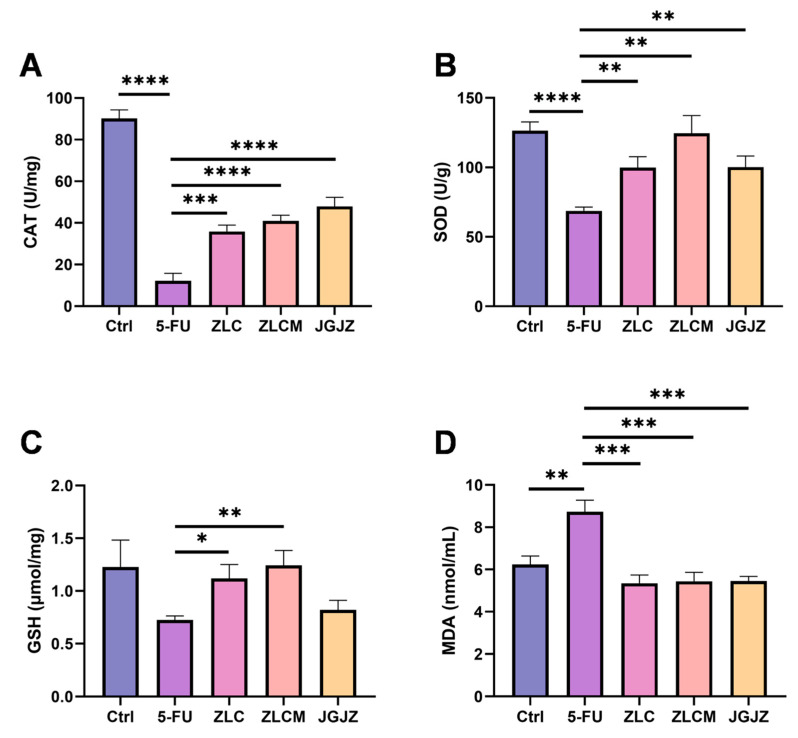
CFBs attenuated intestinal oxidative stress injury in mice induced by 5-FU. Ctrl: the control group; 5-FU: the model group; ZLC: pretreatment with ZLC; ZLCM: pretreatment with ZLCM; JGJZ: the positive control (pretreatment with JGJZ). (**A**) CAT; (**B**) SOD; (**C**) GSH; (**D**) MDA. Data are presented as mean ± SEM, and differences were regarded as statistically significant at a *p*-value < 0.05 (* *p* < 0.05, ** *p* < 0.01, *** *p* < 0.001, or **** *p* < 0.0001).

Oxidative stress, caused by free radicals, contributes significantly to aging and disease. It can impair intestinal barrier integrity by affecting a number of physiological processes, such as reducing antioxidant secretion, increasing TJ disruption, and slowing intestinal epithelial cell regeneration [36]. 5-FU can stimulate oxidative stress during treatment, which damages intestinal epithelial cells in the gastrointestinal system [9]. These damaged cells send out signals that set off pro-inflammatory reactions, further causing gastrointestinal generalized mucositis [12,37]. Markers of oxidative damage in small intestine tissues, including CAT, SOD, GSH, and MDA, were measured. 5-FU administration substantially lowered the contents of CAT (*p* < 0.0001), SOD (*p* < 0.0001), as well as GSH in intestinal homogenates compared to the Ctrl group (Figure 8A–C). The MDA content rose dramatically after 5-FU therapy (*p* < 0.01) (Figure 8D). In contrast, supplementing with CFBs considerably improved the decrease in intestinal CAT (*p* < 0.0001), SOD (*p* < 0.01), and GSH (*p* < 0.01) contents caused by the 5-FU injection, enhancing intestinal oxidative damage resistance, thus significantly decreasing the MDA level (*p* < 0.001). Moreover, the ZLCM group had higher levels of GSH and CAT than the ZLC and JGJZ groups.

### 3.8. CFB Inhibited 5-FU-Induced Intestinal Inflammatory Response

**Figure 9:**
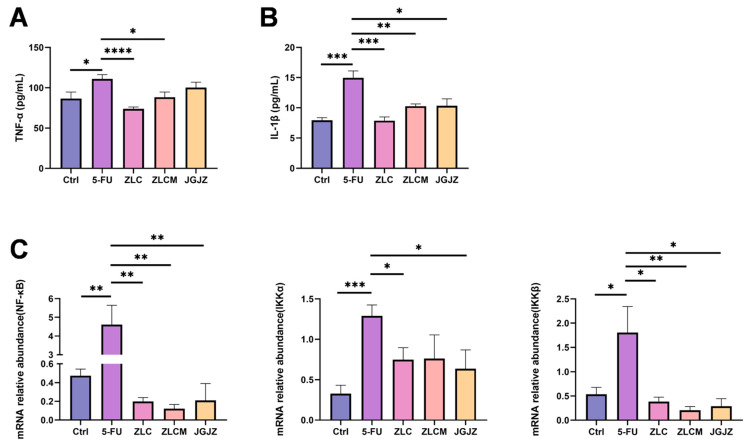
CFBs inhibited 5-FU-induced intestinal inflammatory response. Ctrl: the control group; 5-FU: the model group; ZLC: pretreatment with ZLC; ZLCM: pretreatment with ZLCM; JGJZ: the positive control (pretreatment with JGJZ). Contents of pro-inflammatory cytokines (**A**) TNF-α and (**B**) IL-1β; (**C**) mRNA expression levels of NF-κB, IKKα, and IKKβ. Data are presented as mean ± SEM, and differences were regarded as statistically significant at a *p*-value < 0.05 (* *p* < 0.05, ** *p* < 0.01, *** *p* < 0.001, or **** *p* < 0.0001).

This study demonstrated that 5-FU could stimulate intestinal oxidative stress and cause severe oxidative damage. Numerous studies have demonstrated that oxidative stress increases paracellular permeability, decreases transepithelial electrical resistance, and reduces TJ expression or translocation. Such disturbance can cause gastrointestinal inflammation and possibly a systemic inflammatory reaction [10,38]. Thus, the inflammatory factors in the small intestine were assessed by using the ELISA method. As shown in Figure 9A,B, the 5-FU group’s TNF-α and IL-1β activities were considerably increased (*p* < 0.05 and *p* < 0.001). Nevertheless, CFB treatment, including ZLC (*p* < 0.0001, *p* < 0.001) and ZLCM samples (*p* < 0.05, *p* < 0.01), significantly attenuated this increase and was better than JGJZ.

Furthermore, the mRNA expression levels of NF-κB, IKKα, and IKKβ were assessed to investigate the mechanism of the inflammatory response triggered by 5-FU. The reverse transcription-qPCR research revealed that, in comparison to normal mice, NF-κB, IKKα, and IKKβ levels were markedly activated (*p* < 0.01, *p* < 0.001, and *p* < 0.05, respectively). However, the administration of CFBs alleviated these changes (*p* < 0.01, *p* < 0.05, and *p* < 0.05, respectively) (Figure 9C), suggesting that CFBa could protect intestinal tissues against 5-FU-induced inflammatory response. Therefore, it is reasonable to speculate that 5-FU treatment promotes the release of ROS, inducing intestinal oxidative stress and severe oxidative damage. IL-1β and TNF-α are released as a result of this disturbance, which in turn contributes to activating the NF-κB inflammatory pathway and inducing the subsequent cascade of inflammatory responses. However, CFB pretreatment can prevent ROS generation and NF-κB pathway activation, thereby improving intestinal barrier dysfunction and oxidative damage caused by 5-FU.

## 4. Discussion

5-FU is a chemotherapeutic medication that is frequently used to treat cancer. However, it can lead to intestinal oxidative damage. Recent studies have demonstrated that fermented cereal-based products exhibit strong anti-inflammatory and anti-oxidative properties. For example, lactic acid bacteria-fermented sourdoughs have been shown to contain 25 antioxidant peptides. When applied to mouse fibroblasts, all of the purified fractions demonstrated antioxidant activity. This is consistent with our findings [39]. Additionally, it has been reported that probiotic fermentation substantially raises the amounts of γ-aminobutyric acid, flavonoids, and polyphenols in rice buckwheat. In mice administered a high-fat diet, this therapy successfully reduced oxidative stress, chronic inflammation, and dyslipidemia [40]. Similarly, in mice administered a Western-style diet, fermented whole-grain quinoa and fermented black barley have shown potential in ameliorating intestinal dysbiosis, demonstrating the ability to regulate specific metabolites and the gene expressions linked to inflammatory and metabolic pathways [41]. Additionally, considerable research has shown that fermented herbal medications may modulate the host immune system, altering the gut microbiota to promote intestinal health [24,42,43,44]. Therefore, we introduced commercial lactic acid bacteria and yeast strains to ferment cereals and herbal medicines (CFBs). We subsequently investigated the antioxidant activity of CFBs and their defenses against intestinal injury in BALB/c mice brought on by 5-FU and its potential mechanisms.

Our results demonstrate the efficient degradation of protein components in cereals and an enrichment in peptide and phenolic substance levels following fermentation. Interestingly, the degree of protein degradation and peptide production in the ZLC sample was significantly greater than that in the other samples, which may be attributed to the antibacterial components in the herbal medicine that inhibited the strain’s protein metabolism [45]. When herbal medicine and cereals were fermented together, acid production was weak, resulting in relatively high pH values and inhibited protein metabolism [46], which affected the release of active peptides and bound polyphenols. Consequently, the types of antioxidant peptides were reduced. These findings suggest potential applications of CFBs in preventing and alleviating intestinal oxidative damage caused by chemotherapy.

5-FU administration was then used to create a mouse model of gastrointestinal oxidative injury, and the effects of the CFBs were assessed in vivo. It can be seen that the hair surrounding the anus and buttocks of the mice treated by 5-FU was clearly contaminated, while the intervention groups improved the symptoms significantly, which was consistent with the previous research [34]. In addition, the small intestine, a key organ for nutrient absorption and homeostasis, was noticeably shorter in the 5-FU group. Nonetheless, the intestinal injury brought on by 5-FU was successfully reduced in the ZLC and ZLCM groups [47].

The intestinal barrier is a crucial mucosal defense that, in addition to digesting and absorbing nutrients, also protects against toxic substances, pathogens, antigens, and other harmful entities. DAO activity, along with the expression of TJs, is strongly connected with the production of nucleic acids and proteins in intestinal tissues [48]. After CFB treatment, the intestinal mucosal barrier was restored, as evidenced by the rise in TJs expression and DAO levels.

The alteration of intestinal integrity and structure is another significant aspect of intestinal damage, leading to villus flattening, inflammatory cell infiltration, and cellular damage [30,49]. In this study, HE staining revealed severe structural damage to the ileal mucosa. Mice in the ZLC and ZLCM groups had similar crypt depth and villus length to the Ctrl group, indicating less intestinal injury. Additionally, goblet cells, which are crucial for mucin production and epithelial protection, were significantly decreased in number after 5-FU therapy [50]. However, mice administered CFBs (ZLC and ZLCM groups) had significantly more goblet cells, and the content of Mucin-2 was also increased following CFB pretreatment. These findings suggest that the oral administration of CFBs can attenuate mucosal damage in inflamed mice caused by 5-FU.

Previous studies have also shown that Bcl-2 family proteins contribute to redox regulation. Overexpressing Bcl-2 can prevent cells from oxidative damage induced by H_2_O_2_ or menadione [51], thereby reducing inflammation by inhibiting apoptosis and enhancing antioxidant defenses. This study showed that pretreatment with CFBs could substantially increase the mRNA expression of Bcl-2 while decreasing the expression of the apoptosis-related marker Bax.

Oxidative stress occurs when the overproduction of reactive oxygen species in tissues or cells overwhelms the defense mechanism, leading to tissue or cell damage. Previous studies have established that 5-FU often induces ROS overproduction and stimulates oxidative stress in the gastrointestinal tract when used for anticancer therapy [7]. Moreover, MDA can increase the generation of ROS, which will further damage the intestinal mechanical barrier and reduce intestinal mucosal permeability [52]. According to our findings, the mice that received only 5-FU injections had abnormally elevated MDA levels and lower small intestinal SOD, CAT, and GSH levels, while the intervention of CFBs could successfully mitigate the intestinal oxidative damage after 5-FU treatment. Indeed, it has been proved that ROS could stimulate the up-regulation of pro-inflammatory cytokines [53]. Therefore, in addition to identifying inflammatory markers, this study discovered that the small intestine had greatly higher levels of TNF-α and IL-1β. To further elucidate the molecular mechanism, NF-κB and IKKα/β mRNA expression levels were ascertained in this study. Our research indicates that the small intestine cells in the 5-FU group had substantially higher levels of NF-κB, IKKα, and IKKβ mRNA expression, while pretreatment with CFBs significantly inhibited their abnormal activation.

Based on the above findings, it is reasonable to speculate that 5-FU injection causes intestinal oxidative stress, which activates the NF-κB inflammatory pathway. However, CFB pretreatment successfully prevents aberrant NF-κB pathway activation and guards against intestinal mucosal damage brought on by 5-FU, characterized by increasing tight junctions and reducing apoptosis in intestinal cells. These protective effects may be attributed to the high antioxidant activity of CFBs, which could enhance the activities of antioxidant enzymes (SOD, CAT, and GSH) and decrease the levels of inflammatory and oxidative damage markers (IL-1β, TNF-α, and MDA). In summary, these results imply that CFBs can alleviate the intestinal damage caused by 5-FU by attenuating intestinal oxidative stress and inhibiting the activation of inflammatory pathways.

Although our research has demonstrated the potential use of CFBs as a therapeutic agent against intestinal mucositis, many research limitations remain. We still need to explore the efficacy of CFBs in different chemotherapy conditions through long-term clinical trials and further determine the dosage of CFBs for people with different health conditions, thus achieving the precise nutritional intervention with CFBs for chemotherapy-induced intestinal mucositis.

## 5. Conclusions

In conclusion, our findings indicate that the CFBs contain high levels of polyphenols, flavonoids, and antioxidant peptides and exhibits significant free radical scavenging capacity. Meanwhile, this study has shown that CFBs could increase intestinal tight junctions, reduce cell apoptosis, and maintain the integrity of the intestinal mucosal barrier, thus effectively ameliorating 5-FU-induced intestinal epithelial damage. Additionally, our results indicate that CFBs mitigates 5-FU-induced intestinal injury by attenuating intestinal oxidative stress and inhibiting intestinal inflammation response. Notably, this study provides a new strategy for maintaining intestinal health during chemotherapy-induced mucositis. Moreover, the further exploration of the efficacy of CFBs in different chemotherapy states by long-term clinical trials is valuable for achieving precise nutritional intervention for chemotherapy-induced mucositis.

## Figures and Tables

**Table 1:** Primer sequences.

Gene	Forward Primer	Reverse Primer
ZO-1	GCGGTGCTTTAGCGAACAGAAGGAG	ACCAGTGTGACCTTGGTGGGCTTTG
Occludin	AGGGCTCTTTGGAGGAAGCCTAAAC	AACAGGAAGCCTTTGGCTGCTCTTG
Mucin-2	AAGGGCTCGGAACTCCAGAAAGAAG	TGTAATCACAGAGGCCAGGGAATCG
Bax	GGATGCGTCCACCAAGAAG	CAAAGTAGAAGAGGGCAACCAC
Bcl-2	GACCGCGTATCAGAGCTTTGAGCAG	CCTTGTCTACGCTTTCCACGCACAG
NF-κb	GATTTCCTCCCTACGGTGGGATTAC	CAGACTCTCCTCGTCATCACTCTTG
IKKα	TGTAAAGGCCTGTGATGTCCCTGAG	GTTTGTTGAGTAGCTTCCGGAGGTC
IKKβ	AGTACACCGTGACCGTTGACTACTG	CAACGATGTCCACTTCGCTCTTCTG
β-actin	ACAGCAGTTGGTTGGAGCAA	ACGCGACCATCCTCCTCTTA

## Data Availability

The original contributions presented in the study are included in the article; further inquiries can be directed to the corresponding author.

## Supplementary Materials

The following supporting information can be downloaded at: https://www.mdpi.com/article/10.3390/nu16244332/s1, Table S1: The antioxidant peptide types of CFBs.
